# Role of cytokines in photodynamic therapy-induced local and systemic inflammation

**DOI:** 10.1038/sj.bjc.6600864

**Published:** 2003-05-27

**Authors:** S O Gollnick, S S Evans, H Baumann, B Owczarczak, P Maier, L Vaughan, W C Wang, E Unger, B W Henderson

**Affiliations:** 1 PDT Center, Roswell Park Cancer Institute, Elm and Carlton St, Buffalo, NY 14263, USA; 2 Department of Immunology, Roswell Park Cancer Institute, Elm and Carlton St, Buffalo, NY 14263, USA; 3 Department of Molecular and Cellular Biology, Roswell Park Cancer Institute, Elm and Carlton St, Buffalo, NY 14263, USA

**Keywords:** photodynamic therapy, inflammation, cytokines, chemokines, adhesion molecules

## Abstract

Photodynamic therapy (PDT) of tumour results in the rapid induction of an inflammatory response that is considered important for the activation of antitumour immunity, but may be detrimental if excessive. The response is characterised by the infiltration of leucocytes, predominantly neutrophils, into the treated tumour. Several preclinical studies have suggested that suppression of long-term tumour growth following PDT using Photofrin® is dependent upon the presence of neutrophils. The inflammatory pathways leading to the PDT-induced neutrophil migration into the treated tumour are unknown. In the following study, we examined, in mice, the ability of PDT using the second-generation photosensitiser 2-[1-hexyloxyethyl]-2-devinyl pyropheophorbide-a (HPPH) to induce proinflammatory cytokines and chemokines, as well as adhesion molecules, known to be involved in neutrophil migration. We also examined the role that these mediators play in PDT-induced neutrophil migration. Our studies show that HPPH-PDT induced neutrophil migration into the treated tumour, which was associated with a transient, local increase in the expression of the chemokines macrophage inflammatory protein (MIP)-2 and KC. A similar increase was detected in functional expression of adhesion molecules, that is, E-selectin and intracellular adhesion molecule (ICAM)-1, and both local and systemic expression of interleukin (IL)-6 was detected. The kinetics of neutrophil immigration mirrored those observed for the enhanced production of chemokines, IL-6 and adhesion molecules. Subsequent studies showed that PDT-induced neutrophil recruitment is dependent upon the presence of MIP-2 and E-selectin, but not on IL-6 or KC. These results demonstrate a PDT-induced inflammatory response similar to, but less severe than obtained with Photofrin® PDT. They also lay the mechanistic groundwork for further ongoing studies that attempt to optimise PDT through the modulation of the critical inflammatory mediators.

Photodynamic therapy (PDT) can efficiently and rapidly eradicate local tumours, leading to palliation of advanced disease or cure of early disease (Dougherty *et al*, 1998). The tumour response to PDT involves a complex interplay between direct cytotoxicity to the tumour cells and secondary damage to the tumour and adjacent tissue. In PDT employing the photosensitiser Photofrin®, the combined effects on these tissue targets result in an intense inflammatory response and tumour involution within 24 h. The inflammatory response is considered an important priming event for the development of specific antitumour immunity associated with Photofrin® PDT (Korbelik, 1996; Korbelik and Cecic, 1998; Dougherty *et al*, 1998). Photofrin®-PDT-induced inflammatory changes are characterised by enhanced expression of a number of proinflammatory cytokines, including interleukin (IL)-1*β*, tumour necrosis factor (TNF)-*α* and IL-6 (Evans *et al*, 1990; Nseyo *et al*, 1990; Kick *et al*, 1995; Gollnick *et al*, 1997). PDT-induced infla-mmation is accompanied by leucocyte infiltration into the treated tumour. A major fraction of the infiltrating cells are neutrophils, but also included are mast cells and monocytes/macrophages (Korbelik *et al*, 1996; Gollnick *et al*, 1997). Experimental depletion of neutrophils through the use of anti-GR1 monoclonal antibodies (mAb) diminishes the tumour response to Photofrin®-PDT (de Vree *et al*, 1996a; Korbelik *et al*, 1996; Korbelik and Cecic, 1999). Based on these studies, it has become widely accepted that activated neutrophils in the treated tumour and their cellular functions are an important component in achieving long-term suppression of tumour growth following PDT. However, while the inflammatory response may be important to stimulate an adaptive antitumour response, excessive PDT-induced inflammation can cause severe adverse effects clinically (Dougherty, 2002).

The specific inflammatory pathways stimulated by PDT in tumour tissues *in vivo* have not been well defined. Recruitment of blood-borne neutrophils to sites of infection or tissue damage is tightly controlled by the locally produced proinflammatory cytokines and chemokines. Therefore, it is predicted that the same mechanisms are likely to be involved in PDT-induced inflammatory responses. TNF-*α*, IL-1*β* and IL-6, produced by resident macrophages and stromal cells following stimulation, enhance the expression of vascular adhesion molecules including E-selectin and intracellular adhesion molecule (ICAM)-1 (Butcher and Picker, 1996; Di Carlo *et al*, 2002) as well as the synthesis of chemokines such as IL-8 (in humans, or macrophage inflammatory protein (MIP-2) in rodents) and Gro-*α* (in humans, or KC in rodents) (Mackay, 2001). These molecules collaboratively support the stepwise adhesion cascade required for neutrophil extravasation.

The effort to optimise PDT treatments has generated a number of new photosensitisers that are designed to overcome the problem of prolonged skin phototoxicity present with Photofrin®. One of these, 2-[1-hexyloxyethyl]-2-devinyl pyropheophorbide-a (HPPH) (Bellnier *et al*, 1993), is a highly effective second-generation photosensitiser currently undergoing clinical testing. While this agent, upon light activation, provokes both direct tumour cell toxicity and vascular responses very similar to Photofrin® (Henderson *et al*, 1997), we have observed that the tumour response differs markedly from that following Photofrin®-PDT. The inflammatory changes appear milder upon macroscopic observation and tumour involution occurs over a prolonged period of 48–96 h as opposed to acute effects of Photofrin®-PDT that occur within hours. Keeping in mind the balance between an inflammatory response sufficient to activate antitumour immunity on the one hand, and an excessive response that raises the danger of adverse effects, we decided to examine the inflammatory response evoked by tumour treatment with the new photosensitiser HPPH. This work represents the first detailed mechanistic analysis of the involvement of proinflammatory cytokines, chemokines and adhesion molecules in the inflammatory response to PDT.

## MATERIAL AND METHODS

### Animals and tumour system

Pathogen-free BALB/cJ and C. 129S2(B6)-Cmkar2^tm1Mwm^ (CXCR2 KO) mice were obtained from the Jackson Laboratories (Bar Harbor, ME, USA) and were used for all experiments. Animals were housed in microisolator cages in a laminar flow unit under ambient light. Six to 12-week-old animals were inoculated intradermally on the shoulder with 2 × 10^5^ EMT6 mammary tumour cells (Henderson *et al*, 1985) harvested from exponentially growing cultures. While the EMT6 cell line is highly antigenic and significantly immunogenic (Kurt *et al*, 1995), no spontaneous tumour regressions were observed in this study. Prior to tumour inoculation and/or light treatment, all hair was removed from the prospective treatment site by shaving and depilation. Tumours were used for experimentation about 10 days after inoculation when they had reached a size of 6–8 mm in diameter. All animal experimentation was carried out following ethical committee approval and meet the standards required by the UKCCCR guidelines (Workman *et al*, 1998).

### Reagents

Clinical-grade, pyrogen-free HPPH was obtained from the Roswell Park Pharmacy and reconstituted to 0.4 mM in pyrogen-free 5% dextrose (D5W; Baxter Corp., Deerfield, IL, USA) containing 2% ethanol and 0.1% Tween. Antibodies against murine KC (rat IgG_2a_), MIP-1 (rat IgG_2b_), MIP-2 (rat IgG_2b_) and IL-6 (goat polyclonal IgG) were purchased from R&D Systems (Minneapolis, MN, USA). Rat IgG and goat IgG were obtained from Caltag (San Francisco, CA, USA) and used as isotype controls. Antimurine E-selectin (CD62E) mAb (10E9.6, rat IgG_2a_*κ*), antimurine ICAM-1 (CD54) mAb (3E2, Armenian hamster IgG_1_*κ*) and RITC-labelled murine antihamster IgG (G70-204, G94-90.5) were purchased from BD Pharmingen (San Diego, CA, USA). Antimurine PECAM-1 (CD31) mAb (390, rat IgG_2a_*κ*) was from Beckman Coulter (Miami, FL, USA). Goat anti-rat IgG-FITC, rat serum and hamster sera were from Sigma, Inc. (Saint Louis, MO, USA). Goat serum was a gift from Dr Richard Bankert (State University of New York at Buffalo, Buffalo, NY, USA) and mouse serum was from Dr Yasmin Thanavala (Roswell Park Cancer Institute, Buffalo, NY, USA).

### *In vivo* PDT treatment

Animals were given intravenous injections via tail vein of 0.6 *μ*mol kg^−1^ HPPH, followed 24 h later with illumination at 665 nm light using an argon-dye laser system (Spectra Physics, Mt View, CA, USA). A treatment field 1 cm in diameter, containing the tumour, was illuminated at 75 mW cm^−2^ for a total light dose of 100–135 Jcm^−2^. In some experiments, a single tumour was treated on animals bearing tumours on both shoulders; the untreated or contralateral tumour was used as a ‘drug only’ control. Following PDT, animals were either observed for tumour regrowth, or were killed and tumours were harvested at selected time intervals following light exposure for cell and protein analysis, and immunohistochemistry. In some experiments, animals were bled by tail-end clipping when consecutive daily blood samplings were needed or via heart puncture at the end of experiments. Serum from these blood samples were subjected to immunoelectrophoretic quantification of haptoglobin (HP) (Baumann, 1988) or ELISA. All experiments included control untreated animals and treated animals with photosensitiser only. All animal experimentation was carried out following ethical committee approval and meet the standards required by the UKCCCR guidelines (Workman *et al*, 1998).

### ELISA

Tumour tissues were processed immediately after harvest as described (Gollnick *et al*, 1997). Total protein was determined by the Bio-Rad protein assay (Bio-Rad Lab., Hercules, CA, USA). KC, MIP-1, MIP-2 and IL-6 protein levels in control and treated tumours, and/or serum were determined by ELISA. ELISA kits specific for each protein were purchased from R&D Systems and used according to the manufacturer's suggestion. The assays were performed in triplicate on samples isolated from three animals.

### Flow cytometry

The cell populations present in EMT6 tumours before and after PDT were characterised through FACS analysis, using panels of mAbs to detect specific cell surface antigens as described previously (Gollnick *et al*, 1997). mAbs conjugated directly with fluorescein or phycoerythrin or biotin were used to quantify cells expressing the common leucocyte antigen CD45 (GIBCO/BRL), CD4 and CD8 T-cell antigens (PharMingen, San Diego, CA, USA), CD11b (PharMingen), IA^d^ (PharMingen) and Gr-1 (PharMingen). Appropriate immunoglobulin isotypes were used as controls. In cases where biotinylated antibodies were used, streptavidin-cychrome (PharMingen) was added as a detection reagent.

For flow cytometric analysis, a two-laser FACStar Plus (Becton-Dickinson, San Jose, CA, USA) flow cytometer was used, operating in the ultraviolet (UV) and at 488 nm. Four colours and light scattering properties could be resolved employing 420/20, 530/30 and 575/30 band-pass filters and a 640 long-pass filter. Data were acquired from 5000 cells, stored in collateral list mode, and analysed using the WinList processing program (Verity Software House, Inc., Topsham, ME, USA). Results are presented as the average percentage of total cells; a total of three animals were analysed for each treatment group.

### Immunofluorescence analysis of vascular adhesion molecules

EMT6 murine mammary tumours were snap frozen in Tissue Tek (Sakura, Torrance, CA, USA) and 9 *μ*m cryostat sections were fixed for 10 min using a 3 : 1 methanol/acetone solution. For immunofluorescence staining, tissue sections were sequentially washed in 0.02% PBS azide and 0.1% PBS Triton X-100 before blocking with 10% goat serum or 10% mouse serum diluted in a 1% fetal bovine serum/RPMI 1640 solution (GIBCO BRL, Grand Island, NY, USA) for 10 min. The sections were then incubated for 1 h with mAbs specific for murine E-selectin, ICAM-1 or PECAM-1. As controls, sections were incubated with either rat sera or hamster sera (Sigma, Inc., Saint Louis, MO, USA). The sections were washed for 1 h in 0.02% PBS azide and then incubated with either FITC or RITC-labelled secondary Ab for 1 h. The sections were then washed overnight in 0.02% PBS azide and mounted using Aqua Poly/Mount (Polysciences, Inc., Warrington, PA, USA). Images were recorded using an Olympus BX50 upright microscope equipped with a SPOT RT camera (Spectra Services, Webster, NY, USA) with equivalent exposure times and image settings.

### Neutrophil isolation and frozen-section adhesion assay

Human peripheral blood neutrophils were isolated from normal donor buffy-coat leucocyte concentrates (American Red Cross, Rochester NY, USA) by Ficoll – Hypaque centrifugation and 3% dextran/0.9% saline-sedimentation as described (Anderson *et al*, 1981; Smith *et al*, 1989). Following hypotonic lysis of red blood cells (RBC) in 0.2% saline, isolated neutrophils (>99% pure) were suspended at 5 × 10^7^ ml^–1^ in 1% fetal calf serum (FCS)/RPMI 1640 (GIBCO BRL) and used immediately in adhesion assays.

Adhesion of isolated neutrophils to tumour microvessels in frozen-tissue sections was assessed *in vitro* under mechanical shear as described (Lewinsohn *et al*, 1987; Evans *et al*, 2001). Briefly, a total of 5 × 10^6^ neutrophils in 100 *μ*l of FCS/RPMI 1640 medium were overlaid onto 12 *μ*m cryosections of tumour tissues from PDT-treated or nontreated mice. Selected tumour tissue specimens were pretreated with function-blocking mAb specific for E-selectin (10 *μ*g ml^−1^; BD Pharmingen, San Diego, CA, USA) or isotype-matched negative control antibodies. The assay was performed at 4°C for 30 min with mechanical rotation (112 r.p.m.; Labline Instrument, Labline Instrument, Inc., Melrose Park, IL, USA). After removal of nonadherent cells, sections were fixed in 3% glutaraldehyde, and stained with 0.5% toluidine/absolute ethanol. Neutrophil adhesion was quantified by light microscopy in a total of 100 microvessels per tumour tissue specimen; data are the mean ±s.e. of triplicate specimens.

### Antibody treatments

For chemokine, cytokine and adhesion molecule neutralisation studies, anti-KC, anti-MIP-2, anti-IL-6, anti-E-selectin or isotype-matched control antibodies were administered immediately following (100 *μ*g mouse^−1^; i.v.) PDT treatment or 24 and 48 h (50 *μ*g mouse^−1^; i.v.) post-PDT treatment. Neutrophil depletion was accomplished using anti-GR-1 antibodies (100 *μ*g mouse^−1^; i.v.) administered 24 h prior to PDT, immediately and 24 h post-PDT. Tumour growth was monitored as above.

### Statistical analysis

Statistical analysis was performed using a nonpaired Student's *t*-test. Statistical analysis of survival data was performed by log-rank test. In all cases, significance was defined as *P*< 0.05.

## RESULTS AND DISCUSSION

### HPPH-PDT increases the proportion of neutrophils in EMT6 tumours

**Figure 1:**
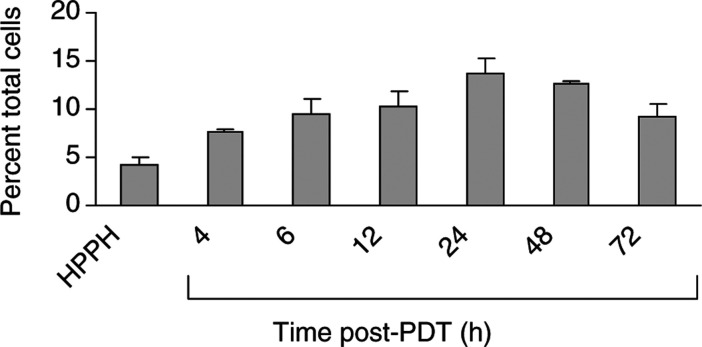
HPPH-PDT enhances neutrophil infiltration into the treated tumour. Animals were treated with 0.6 *μ*mol kg^−1^ HPPH, followed 24 h later by 100 J cm^−2^ of 665 nm light. Tumours were harvested at various times post-PDT and the infiltrating cell populations were analysed by flow cytometry as described in Materials and Methods. Samples were collected from control animals (no treatment, HPPH alone) at 6 h ‘post-PDT’. Results are reported as the percentage of total cells present in the tumour that are CD45^+^ and Gr-1^+^. A minimum of three mice were analysed at each time point. Error bars represent the s.e.

We and others have previously shown that the local inflammatory response following PDT with Photofrin® is characterised by a strong, time-dependent infiltration of neutrophils into the treated tumour (Korbelik *et al*, 1996; Gollnick *et al*, 1997; Sun *et al*, 2002). To determine whether HPPH-PDT also resulted in inflammatory cell infiltration, tumour-infiltrating host cell numbers and phenotype were analysed by flow cytometry. Two phenotypes were predominant among CD45^+^ host cells, neutrophils (CD11^+^ GR1^hi^) and macrophages (CD11^+^GR1^lo^). HPPH-PDT, at a dose that achieved long-term tumour suppression in ∼50% of animals, resulted in a modest, time-dependent increase in the percentage of neutrophils in the treated tumour (Figure 1

). Within 4 h of treatment, the percentage of neutrophils was significantly increased (*P*<0.007 as compared to HPPH treatment in the absence of light). The proportion of neutrophils continued to increase and remained elevated for at least 72 h post-treatment. The differences in neutrophil levels over the period of 6, 12, 24 and 48–72 h postillumination were not significant (*P*>0.41). Moreover, the increase was no more than three-fold over controls and rarely exceeded 20% of total cells during the period of observation. The percentage of macrophages (9.3%) did not change significantly throughout the observation period. The level of lymphocytes did not exceed 5% of total cells and that also did not change significantly after HPPH-PDT (data not shown).

Thus, like Photofrin®-PDT, HPPH-PDT results in an influx of neutrophils into the treated tumour. The kinetics of neutrophil migration observed were similar to those seen following Photofrin®-PDT (Gollnick *et al*, 1997), although the number of infiltrating neutrophils was considerably less following HPPH-PDT (37% of the total cells 24 h following Photofrin®-PDT *vs* 13.8% of the total cells 24 h following HPPH-PDT). These results are consistent with the lower degree of inflammation seen following HPPH-PDT as compared to Photofrin®-PDT (Bellnier *et al*, 1993) and may be a reflection of the kinetics of tumour destruction. Photofrin®-PDT-treated tumours have regressed within the first 24–48 h of treatment, while HPPH-PDT-treated tumours are not eliminated for 48 – 72 h.

### HPPH-PDT enhances expression of neutrophil attractant chemokines in EMT6 tumours

Leucocyte migration is a tightly controlled process that is regulated by chemokines/cytokines and mediated by adhesion molecules. Murine neutrophil migration is regulated in large part by the chemokines MIP-2 and KC. KC is constitutively expressed and is believed to be involved in basal neutrophil migration (Bozic *et al*, 1995). In contrast, MIP-2 is inducible and thought to mediate stress- or injury-induced neutrophil migration (Biedermann *et al*, 2000). Also involved in neutrophilic inflammation is MIP-1 (*α* and *β*), which can activate granulocytes, stimulate the production of reactive oxygen species in neutrophils and induce the generation of proinflammatory cytokines IL-1, IL-6 and TNF (Adams and Lloyd, 1997).

**Figure 2:**
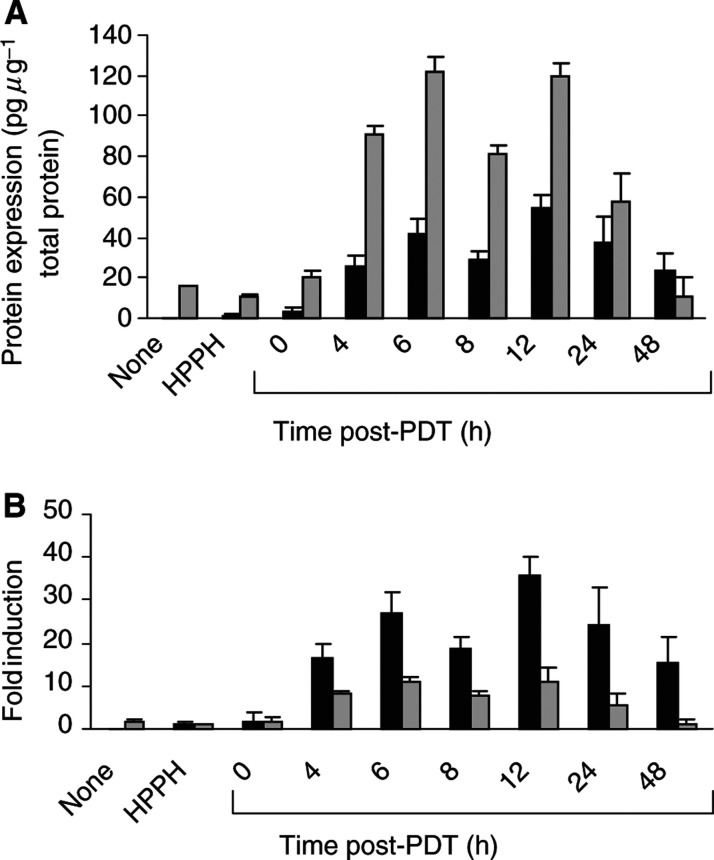
Induction of MIP-2 and KC following PDT. Animals were treated with PDT as described in Figure 1. Tumours and sera were collected from treated animals at various times post-PDT. Tumours and sera were collected from control animals (no treatment, HPPH alone) at 6 h ‘post-PDT’. The total amount of protein per sample was determined using the Bio-Rad protein assay. KC and MIP-2 levels were determined by ELISA and are reported as (**A**) pg *μ*g^−1^ of total protein or (**B**) fold induction where chemokine expression is reported in relation to the expression found in tumours treated with HPPH alone. A minimum of three mice were analysed at each time point. Error bars represent the s.e. MIP-2 
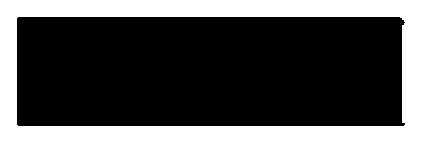
; KC 
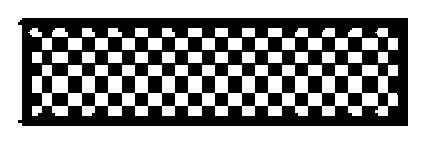

Expression of MIP-2 and KC in HPPH-PDT-treated tumours was significantly elevated by 4 h post-treatment (*P*<0.009 as compared to HPPH controls) and remained elevated for up to 24 h post-treatment. Although the protein levels of KC present in the tumour were higher than those for MIP-2 (Figure 2A

), the fold induction for MIP-2 was significantly higher (*P*<0.01) than that for KC (Figure 2B). The induction of MIP-2 protein by PDT corresponded to a 27.1±4.9-fold induction at 6 h post-treatment as compared to HPPH controls (*P*<0.0004), while the increase in KC corresponded to an 11.2±1.2-fold induction at 6 h post-PDT (*P*<0.0001 when compared to HPPH alone controls). The levels of KC and MIP-2 were unchanged in untreated contralateral tumours as compared to chemokine levels in treated tumours, indicating that the induction of MIP-2 and KC was not the result of a systemic mediator. Thus, HPPH-PDT induced a time-dependent, local increase in two neutrophil attractant chemokines, MIP-2 and KC. The kinetics of expression paralleled that of neutrophil infiltration. We also observed a trend towards increased levels of MIP-1*α* in treated tumours at 24 and 48 h post-treatment (data not shown), but the changes were not significant (*P*<0.167).

### HPPH-PDT induces local and systemic expression of IL-6

**Figure 3:**
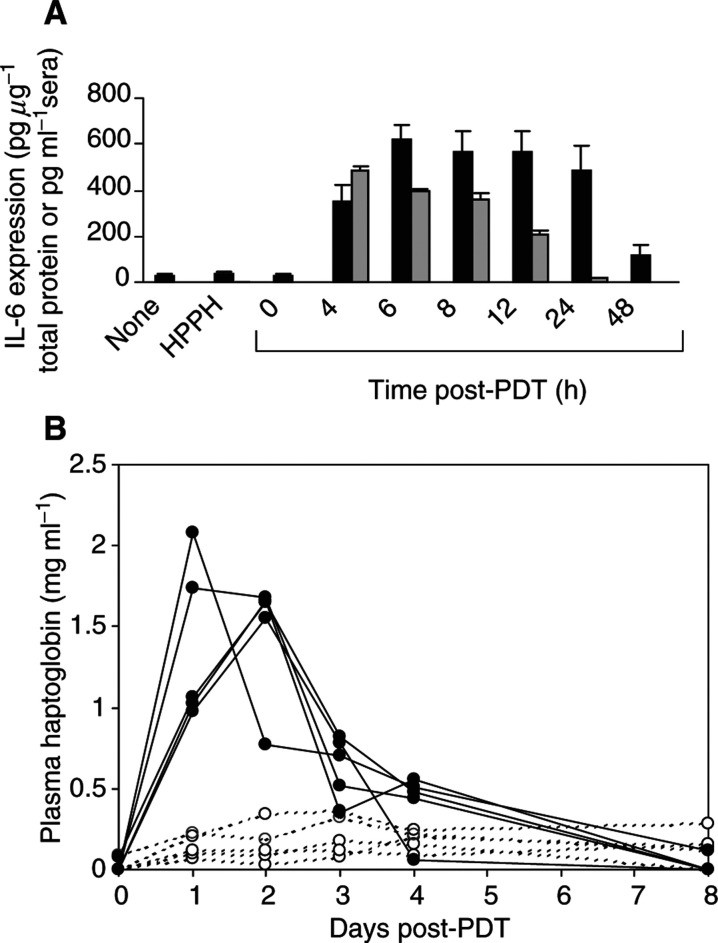
Induction of local and systemic IL-6 by PDT. Samples were collected from control animals (no treatment, HPPH alone) at 6 h ‘post-PDT’. The total amount of protein per tumour was determined using the Bio-Rad protein assay. (**A**) IL-6 levels were determined by ELISA and are reported as either pg *μ*g^−1^ of total protein (tumour) or pg ml^−1^ serum. A minimum of three mice were analysed at each time point. Error bars represent the s.e. Tumour IL-6 
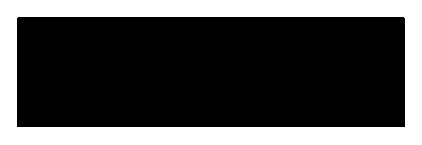
; serum IL-6 
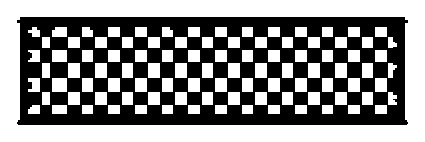
. (**B**) EMT6 tumour-bearing mice were subjected to HPPH treatment. At 0 h, one group of five mice was exposed to light (135 J cm^−2^) (filled symbols), and another group of five mice was kept in the dark (control, open symbols). Every 24 h, a blood sample was collected for each mouse and the serum level of HP was determined by immunoelectrophoresis. The values were calculated in mg ml^−1^ and the HP concentration for each mouse and bleeding was reproduced.

We have previously shown that Photofrin®-PDT stimulates the expression of IL-6 in the tumour (Gollnick *et al*, 1997). IL-6 is characterised as a proinflammatory cytokine primarily because of its role in release of acute-phase proteins (Baumann and Gauldie, 1994; Suffredini *et al*, 1999) and complement activation (Knittel *et al*, 1997; Minta *et al*, 2000; Schieferdecker *et al*, 2000). In some reports, IL-6 has also been implicated in neutrophil migration (Dalrymple *et al*, 1995; Romano *et al*, 1997; Hurst *et al*, 2001; Suwa *et al*, 2001) and has been shown to contribute to the rise in circulating neutrophils observed following Photofrin-PDT (Cecic and Korbelik, 2002). Figure 3A

shows that HPPH-PDT strongly enhanced the expression of IL-6 in the tumour. IL-6 levels rose sharply up to 6 h after treatment (*P*< 0.0001 as compared to HPPH treatment alone) and remained significantly elevated during the 48 h post-treatment period (*P*<0.0003). HPPH-PDT also induced a significant increase in circulating levels of IL-6 (Figure 3A), which became evident within 4 h post-treatment (*P*<0.0008 when compared to HPPH only controls). The levels remained significantly elevated up to 24 h post-treatment (*P*<0.018 as compared to HPPH alone controls). The kinetics of local IL-6 induction reached maximal levels by 6 h post-treatment, which is analogous to the kinetics observed for the chemokines, MIP-2 and KC, as well as for neutrophil migration. Similar kinetics of IL-6 expression were also observed following Photofrin®-PDT (Gollnick *et al*, 1997).

The bioactivity of the systemic IL-6 was determined by the level of haptoglobin (HP) in the sera of control (HPPH only) and HPPH-PDT-treated animals over an 8 day post-PDT treatment period (Figure 3B). HP is a member of the type 2 acute-phase proteins and the transcriptional activation of the Hp gene in liver is dependent upon IL-6 (Baumann *et al*, 1989). The relative change in expression of this acute-phase protein is proportional to the level of inflammation (Baumann and Gauldie, 1994). The sera of animals treated with HPPH-PDT reached peak values of 2 mg ml^−1^ of serum 24–48 h post-PDT. The HP concentration returned to pretreatment levels after 8 days post-treatment period. In contrast, the sera of control animals, which received HPPH but were not exposed to light, maintained an HP level of <0.3 mg ml^−1^ over the course of the experiment. The time course and relative change in circulating HP was characteristic for an acute-phase reaction observed after severe forms of tissue injury, and that involved the mediator function of IL-6 (Kopf *et al*, 1994).

### HPPH-PDT enhances the expression of ICAM and E-selectin in EMT6 microvessels

**Figure 4:**
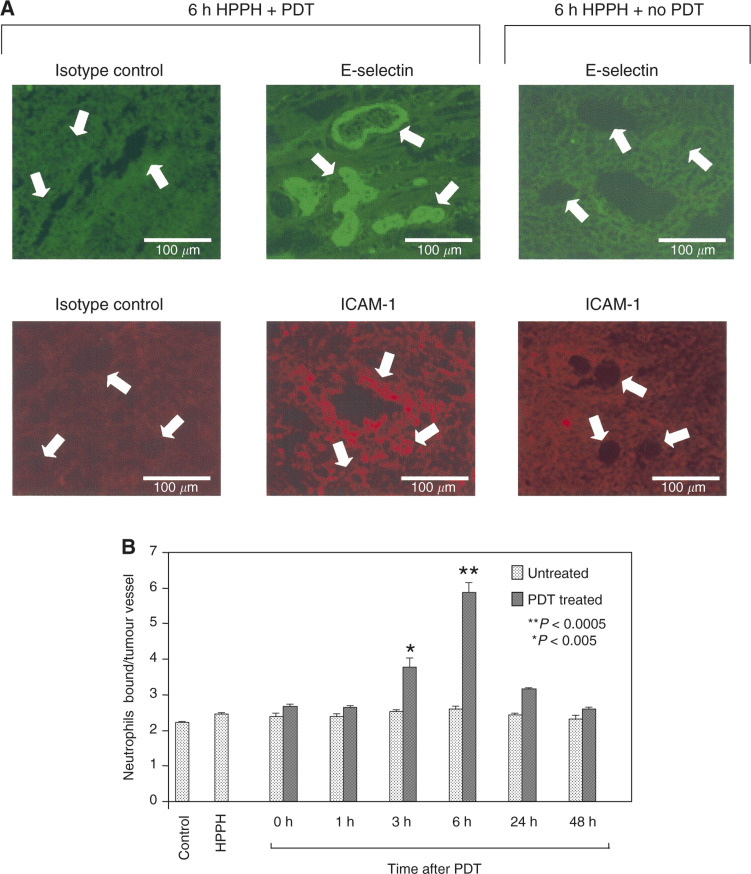
HPPH-PDT enhances adhesion molecule expression and neutrophil adhesion in tumour microvessels. Animals were treated as in Figure 1 for the indicated time periods and tumour cryosections were either stained for vascular adhesion molecule expression using specific mAb (**A**) or evaluated for the ability to support adhesion of neutrophils under mechanical shear (**B**). In (**A**), arrows denote tumour vessels; note that fluorescence was not detected in tumour vessels stained with isotype-matched control Ab (left panels) or in tumours that were not treated with light (i.e. no PDT, right panels). In contrast, E-selectin and ICAM-1 were highly expressed on tumour vessels following HPPH-PDT treatment. Data in (**B**) are the mean +s.d. of triplicate samples and are representative of three independent experiments. The differences between adhesion in untreated contralateral tumours and PDT-treated tumours were significant, *P*<0.005 (^*^), *P*<0.0005 (^**^) by unpaired two-tailed Student's *t-*test.

In addition to a dependence on chemokines, expression of vascular endothelial adhesion molecules is also required for neutrophil migration. The effect of HPPH-PDT on the expression of E-selectin and ICAM-1 was examined by immunofluorescence analysis (Figure 4A

) and the results are summarised in Table 1

**Table 1:** Immunofluorescence staining patterns of adhesion molecules on EMT6 microvessels following PDT

	**Time after PDT (h)**										
**Adhesion molecule**	**None**	**HPPH**	**0**	**1**	**3**	**6 (PDT)**	**6 (Untx)^a^**	**8**	**12**	**24**	**48**
**E-selectin (CD62E)**	−b	−	−/±	−/±	−/+	−/+++	−	−/+	−/±	−/±	−
**ICAM-1 (CD54)**	−/±	−/±	−/+	−/+	−/++	−/±	−/++	−/++	−/+	−/+	−/±
**PECAM-1 CD31**	+++	+++	+++	+++	+++	++++	+++	++	−/++	ND^c^	ND

a Untx refers to the contralateral tumour that was not treated with light in animals receiving HPPH and having one tumour treated with light.

b Staining intensity of tumour microvessels ranges from undetectable (−) to very high levels (++++).

c ND, not determined.

. HPPH-PDT resulted in a transient time-dependent increase in E-selectin and ICAM-1 expression on the tumour microvessels that peaked at 6 h post-treatment and returned to baseline by 48 h post-treatment. PDT-induced expression of E-selectin and ICAM-1 was dependent on the presence of light as adhesion molecule expression did not increase in the untreated contra-lateral tumour. This finding also indicates that circulating cytokines were not effective in eliciting the change in adhesion molecules, but depended on locally produced regulatory factors. The induction of E-selectin and ICAM-1 was heterogeneous, ranging in staining intensity from − to ++ at the 6 h point for example (Table 1). In this regard, some vessels within the tumour showed a marked increase in vascular display of E-selectin while other vessels in close proximity failed to respond (Figure 4A). The increase in E-selectin was restricted to the tumour microvasculature. In contrast, the increase in ICAM-1 immunofluorescent staining was more diffuse, reflecting of enhanced expression on both vascular endothelium and infiltrating cells. Negative control staining with isotype-matched rat IgG or hamster IgG confirmed that E-selectin and ICAM-1staining was specific. The sections were also stained with PECAM-1 (CD31) as a control. PECAM-1 staining was unaffected by PDT at the earlier time points (up to 6 h); however, the staining intensity began to diminish at 8 h post-treatment. This decrease likely reflects a loss in vessel integrity rather than a loss of PECAM-1 expression (BWH and SE, unpublished observations). Like the enhanced expression of chemokines and IL-6, the kinetics of adhesion molecule expression mirrors those of neutrophil influx.

### HPPH-PDT stimulates adhesion of neutrophils to EMT6 microvessels

The effect of PDT on the function of adhesion molecules was monitored by determining the ability of neutrophils to adhere to EMT6 microvessels in frozen-tissue sections under mechanical shear following *in vivo* PDT treatment. As can be seen in Figure 4B, PDT significantly enhanced neutrophil/endothelial adhesion in a time- and light-dependent manner (drug alone *vs* 6 h post-treatment: *P*<0.0005) with kinetics similar to those observed for induction of adhesion molecule and chemokine expression. The enhanced adhesion was restricted to tumours that had been treated with PDT. No increases in neutrophil adhesion were observed in contralateral tumours, which did not receive light, on the same animal, implicating again a local rather than systemic mechanism.

### HPPH-PDT enhanced neutrophil migration is dependent upon E-selectin and MIP-2

**Figure 5:**
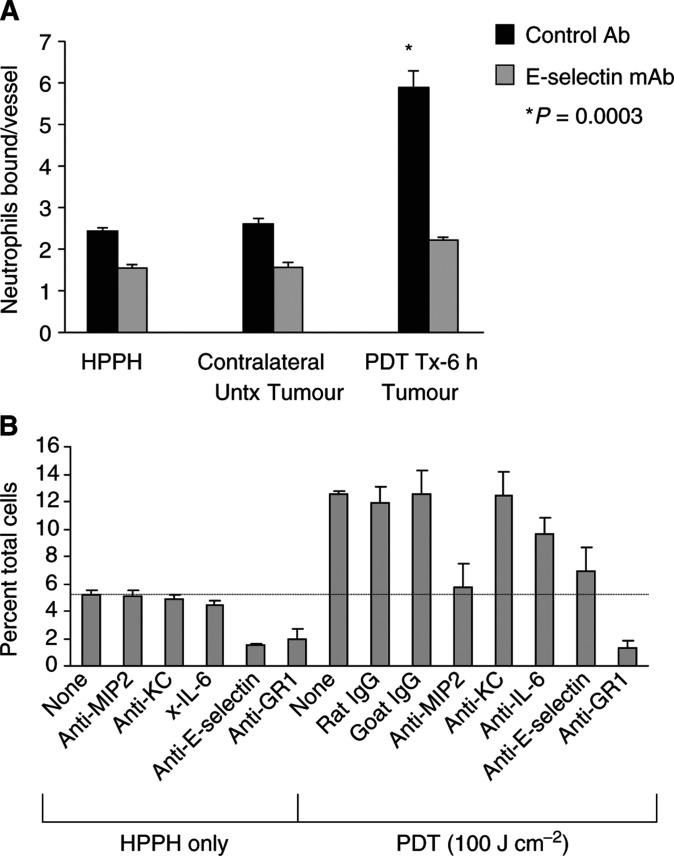
(**A**) HPPH-PDT stimulates E-selectin-dependent adhesion of neutrophils to tumour microvessels. Animals were treated with HPPH for 6 h and separated into two groups: (1) the drug only group did not receive PDT treatment and (2) in the second group, tumours were treated with PDT, while contralateral tumours were not treated with light. Neutrophil adhesion to tumour microvessels was evaluated under mechanical shear in tumour cryosections in the presence of isotype-matched negative control Ab or function-blocking mAb specific for E-selectin (10 *μ*g ml^−1^). Data are the mean ±s.d. of triplicate samples and are representative of three independent experiments. The difference between adhesion in untreated contralateral tumours and PDT-treated tumours was significant, *P* < 0.00035 (^*^) by unpaired two-tailed Student's *t-*test. (**B**) Inhibition of MIP-2 and E-Selectin expression following PDT diminishes PDT-induced neutrophil infiltration into EMT6 tumours. Tumour-bearing animals were treated with PDT as described under Figure 1. Anti-MIP-2, anti-E-selectin, anti-IL-6, anti-GR-1, rat IgG (isotype control for anti-MIP-2 and anti-E-selectin) or goat IgG (isotype control for anti-IL-6 and anti-GR-1) were administered immediately after PDT. Tumours were isolated 24 h post-PDT and the infiltrating cell populations were determined by flow cytometry. The dashed line indicates the level of neutrophils found in control tumours. Mean values from three animals of the percentage of total cells that are CD45^+^ and GR-1^+^ are shown. Error bars represent the s.e.m.

The similarity in kinetics, as well as previous findings (Biedermann *et al*, 2000), led us to postulate that a causal relation may exist between the induction of chemokine/cytokine, functional adhesion molecule expression and neutrophil migration. This hypothesis was confirmed by blocking and depletion studies. HPPH-PDT-enhanced neutrophil adhesion was dependent upon E-selectin as blocking antibodies to E-selectin eliminated the adhesion of neutrophils to EMT6 microvessels (*P*<0.0003; Figure 5A

). Adhesion was not abrogated in the presence of an isotype-matched control antibody. Thus, the characteristic neutrophil infiltration associated with PDT is due, at least in part, to an induction of E-selectin. Other studies have shown that PDT-induced adherence of neutrophils to endothelial cells *in vitro* (de Vree *et al*, 1996b) and *in vivo* (Dellian *et al*, 1995; Adili *et al*, 1996; Sluiter *et al*, 1996; Rousset *et al*, 1999) also involved *β*_2_-integrins (LFA-1) (Sluiter *et al*, 1996; de Vree *et al*, 1996b).

Blocking E-selectin and MIP-2 action through the use of neutralising antibodies, administered immediately after completion of PDT treatment, led to a significant reduction in neutrophil accumulation to almost baseline levels by 6 h post-treatment (PDT+anti-MIP-2 *vs* PDT+rat IgG: *P*<0.0024; PDT+anti-E-selectin *vs* PDT+rat IgG: *P*<0.04) (Figure 5B). This confirms the importance of E-selectin in PDT-induced neutrophil migration, and implies that the increased level of neutrophils in the tumour was the result of recruitment via MIP-2. Administration of anti-GR-1 antibodies also reduced neutrophil levels in both treated and control tumours to levels below those found in when anti-MIP-2 was used (Figure 5B; PDT+anti-GR-1 *vs* PDT+rat IgG: *P*<0.0095). No significant neutrophil reduction was achieved with anti-KC antibodies.

Although IL-6 was shown to affect induction of systemic neutrophilia by Photofrin-PDT (Cecic and Korbelik, 2002; Sun *et al*, 2002), neutralisation of IL-6 did not significantly affect the levels of neutrophils infiltrating the tumour post-PDT (Figure 5B; PDT+anti-IL-6 *vs* PDT+goat IgG: *P*<0.533). Moreover, neutralisation of IL-6 did not block induction of E-selectin or ICAM-1 on tumour microvessels or appear to play a role in long-term control of EMT6 tumours by HPPH-PDT (data not shown). Similar results were recently reported by Sun *et al* (2002). In this study, neutralising antibodies to IL-6 were administered intraperitoneally 30 min prior to mTHPC-PDT of s.c. SCCVII tumours. No effect on long-term tumour response was observed. These results suggest that the role of IL-6 in PDT-induced inflammation may vary depending on the photosensitiser used and the tumour type studied.

The role of MIP-2 in PDT-induced neutrophil migration was confirmed by examining neutrophil migration in mice deficient for CXCR2, the receptor for both MIP-2 and KC (Rollins, 1997), following HPPH-PDT. EMT6 tumours of CXCR2-deficient mice contained very low levels of neutrophils prior to PDT treatment (1.37±0.04% of total cells). HPPH-PDT raised these to 3.98±0.48% of total cells (*P*<0.006). This compares to neutrophil levels of 4.10 ± 0.83% of total cells in untreated tumours *vs* 16.9±3.22% of total cells in HPPH-PDT-treated tumours (*P*<0.018) in wild-type mice.

These results combined indicate that PDT-enhanced neutrophil migration is dependent upon E-selectin and MIP-2, but is not influenced by IL-6. MIP-2 neutralisation has also been shown to reduce neutrophil recruitment in delayed type hypersensitivity responses (Biedermann *et al*, 2000).

In summary, we have shown that HPPH-PDT, like Photofrin® -PDT, initiates a local, albeit less severe, local inflammatory response. It is characterised by an influx of neutrophils into the treated tumour upon the presence of MIP-2 and E-selectin, which are induced locally in a temporal manner following PDT. Neutralisation of these MIP-2 and E-selectin eliminated neutrophil infiltration and increased adhesiveness to tumour microvasculature. Thus, HPPH-PDT-induced inflammation appears to be due, at least in part, to the coordinated induction of chemokine and adhesion molecule expression and activation, which results in the migration of neutrophils into the treated tumour. Once at the tumour site, neutrophils have been implicated in direct tumour cell kill, recruitment of leucocytes and lymphocytes through chemokine/cytokine secretion and tumour rejection in T-cell-dependent reactions (reviewed in Di Carlo *et al*, 2002). In addition, some studies have suggested that neutrophils can also promote malignant growth and progression (Pekarek *et al*, 1995). The results presented here begin to define the mechanisms behind PDT-induced neutrophil migration, which are critical to our understanding of the role these cells play in long-term tumour suppression by PDT. Furthermore, an understanding of the mechanisms leading to PDT-induced inflammation has the potential to provide a means of optimising clinical PDT, possibly through regulation of the mediators of the response. Studies are currently underway to determine whether the HPPH-PDT-induced inflammatory response is involved in the generation of antitumour immunity observed after HPPH-PDT.
